# Antimicrobial Peptides from Plants: A cDNA-Library Based Isolation, Purification, Characterization Approach and Elucidating Their Modes of Action

**DOI:** 10.3390/ijms22168712

**Published:** 2021-08-13

**Authors:** Md. Samiul Islam, Gamarelanbia Mohamed, Shakil Ahmed Polash, Md. Amit Hasan, Razia Sultana, Noshin Saiara, Wubei Dong

**Affiliations:** 1 Department of Plant Pathology, College of Plant Science and Technology and the Key Lab of Crop Disease Monitoring & Safety Control in Hubei Province, Huazhong Agricultural University, Wuhan 430070, China; samiulislam@webmail.hzau.edu.cn (M.S.I.); gameralnbiam@yahoo.com (G.M.); 2 School of Science, RMIT University, Melbourne 3001, Victoria, Australia; shakilpolash.bd.94@gmail.com; 3 Department of Genetic Engineering and Biotechnology, University of Rajshahi, Rajshahi 6205, Bangladesh; amithasan1141@gmail.com; 4 State Key Laboratory of Agricultural Microbiology, Department of Microbiology, College of Life Science and Technology, Huazhong Agricultural University, Wuhan 430070, China; razia@webmail.hzau.edu.cn; 5 Department of Biotechnology and Genetic Engineering, Jahangirnagar University, Savar, Dhaka 1342, Bangladesh; noshinsaiara1993@gmail.com

**Keywords:** antimicrobial peptides, cDNA library, *Bacillus subtilis*, AMP prediction, membrane interrupting peptides

## Abstract

Even in a natural ecosystem, plants are continuously threatened by various microbial diseases. To save themselves from these diverse infections, plants build a robust, multilayered immune system through their natural chemical compounds. Among the several crucial bioactive compounds possessed by plants’ immune systems, antimicrobial peptides (AMPs) rank in the first tier. These AMPs are environmentally friendly, anti-pathogenic, and do not bring harm to humans. Antimicrobial peptides can be isolated in several ways, but recombinant protein production has become increasingly popular in recent years, with the *Escherichia coli* expression system being the most widely used. However, the efficacy of this expression system is compromised due to the difficulty of removing endotoxin from its system. Therefore, this review suggests a high-throughput cDNA library-based plant-derived AMP isolation technique using the *Bacillus subtilis* expression system. This method can be performed for large-scale screening of plant sources to classify unique or homologous AMPs for the agronomic and applied field of plant studies. Furthermore, this review also focuses on the efficacy of plant AMPs, which are dependent on their numerous modes of action and exceptional structural stability to function against a wide range of invaders. To conclude, the findings from this study will be useful in investigating how novel AMPs are distributed among plants and provide detailed guidelines for an effective screening strategy of AMPs.

## 1. Introduction

Antimicrobial peptides (AMPs) are a group of widespread chemical compounds found in nature, produced by various tissues and cells in plants, animals, and invertebrates [1]. AMPs work as a first-line defense in the host immune system and inactivate deleterious bacterial action by disrupting their cell membrane. Furthermore, AMPs regulate the immune system and modulate its inflammatory mechanisms to serve a protective role for host organisms against harmful microbes, including bacteria, fungi, and viruses [2]. AMPs can be found in unpolished soil and saltwater bacteria; multiple techniques are available for generating an enormous library of derivatives [3]. In plants, AMPs can facilitate the immune reaction to protect against various diseases. Besides, when introduced into transgenic plants, synthetic AMPs can serve as bio-pesticides, outweighing the role of chemical pesticides in the agriculture sector [4]. Plant AMPs have unique features, including charge, hydrophilicity, secondary and 3D structure, and distinct conformation of the cysteine residue [5]. Furthermore, in-plant AMPs, a hypervariable nucleotide region exists, which helps to recognize various forms of pathogenic microbes. The cationic charge of these AMPs facilitates the detection of the pathogen’s plasma membrane [6]. Interestingly, plant AMPs possess an amphipathic character that helps take action in both mechanisms: hydrophilic and hydrophobic. The cationic charge and amphipathic nature together enhance the ability to act as a permeability agent in the plasma membrane [7,8]. Furthermore, plant AMPs can be classified into several classes: defensins, heveins, knottins, etc., based on the variable cysteine residue conformation and distinct pattern of disulfide bond [9].

AMPs are composed of 5–50 amino acids and have secondary structures: alpha-helix, beta-sheet, and 3D structure, which make this group more precise against harmful pathogens during immune reaction [10,11]. AMPs show a spatial characteristic—some are expressed constitutively in different parts of a host system. In contrast, others are condition or position-specific, making these AMPs a constant threat for evolving harmful pathogens. Previous studies showed that AMPs function as a ‘cocktail’—a group that usually works together—rather than a single one to maximize their defensive role [12,13,14,15]. Since AMPs possess a positive electrostatic charge, the anionic surface of the microbe’s cell membrane is often vulnerable to AMP actions. Thus, AMPs can attack peptidoglycan, lipopolysaccharide or phosphatidylglycerol, and sphingolipid of gram-positive bacteria, gram-negative bacteria, and fungi, respectively. Injury to these microbes’ outer surface helps AMPs enter inside the cellular level and control their expression system [16,17,18,19]. Moreover, plant AMPs can adapt themselves while fighting against innate immunity. Upregulation of various AMPs is a common scenario, which operates directly through its intricate signaling pathways such as pathogen-associated molecular patterns triggered immunity (PAMPs-PTI), reactive oxygen species (ROS), and effector-triggered immunity (ETI) [20,21]. Furthermore, these AMPs play a significant role in a signaling cascade that prevents the infection/attack of foreign microbes. When any microbes attack the plant body, a stress response is produced, which then turns into a signal. Pattern recognition receptors (PRRs) located in the plant cell surface usually receive these signals. Later, PRRs transfer these signals into the cellular level where various cellular messengers (e.g., ROS, Ca^2+^, NO) accept it and help in the transcriptional reprogramming process that ultimately gives AMPs enough space to take immunological actions to destroy pathogenic microbes. During this time, plant AMPs can modulate their immunological reaction and act accordingly to the types of microbial attack [22,23].

There are several techniques for screening genes, which could be used to search for the genes responsible for antimicrobial peptides: homologous gene sequences [24], PCR amplification and DNA sequencing [25], bDNA (branched DNA) assay [26], PCR-RFLP analysis [27], silicon micro-chip DNA oligonucleotide arrays [28], genome-wide high-throughput screening [29], and fluorescent high-throughput screening [30]. All of these methods have positive sides; however, they possess some major drawbacks—higher costs and lesser screening quality. Therefore, suitable screening techniques for AMPs are needed. At present, *Escherichia coli* and *Bacillus subtilis* expression systems are vastly used [31]. The former is widely popular between these two due to its simple genetic function, low cost, and rapid growth [32]. However, endotoxin removal is a difficult task in the *E. coli* expression system [33]. As a product, it is insoluble, tightly packed, forms denatured inclusion bodies, is biologically inactive, and needs further folding change, solubilization, and downstream purification [34,35]. On the other hand, the *B. subtilis* system releases peptides into its extracellular space and has broad-spectrum antimicrobial gene expression capability [36,37,38,39,40]. The products of this expression system are biologically active and require simple downstream processing [41,42].

Recently, by utilizing the *B. subtilis* expression system, we developed the cDNA library construction method in our laboratory, postulating that this expression system is more robust than the *E. coli* expression system [38]. We found that *B. subtilis* itself produces beneficial antimicrobial compounds and shows potential antimicrobial activity against plant pathogens [43]. Furthermore, the *B. subtilis* expression system possesses some other advantages: no product assembly, better yield, and continuous production and cultivation [44]. Therefore, in this review, a new approach for isolating resistant genes coding for AMPs from the cDNA library via the *B. subtilis* expression system is described and illustrated. Moreover, this study intends to offer guidance to help direct researchers who are involved in isolating, identifying, purifying, and analyzing novel AMPs from plant sources against various classes of pathogens. Finally, excellent knowledge of the mechanism of action of AMPs is also described as an essential factor in the study of more robust and less harmful AMPs.

## 2. Expression Systems Applied for AMP Production

A sufficient number of purified and active molecules are required to efficiently identify a novel AMP. Direct isolation from natural sources, chemical synthesis, and recombinant expression are the three main ways of AMP screening [45]. Most organisms, however, have very low AMP concentrations. As a result, isolating peptides directly from natural sources can be a time-consuming, environmentally unpleasant, and expensive approach. This method can harm the ecosystem, especially when it comes to peptides identified in uncommon creatures with limited populations [46]. Synthetic costs for peptides with disulfide linkages, on the other hand, can be prohibitively high, preventing the production of these proteins [47]. Furthermore, some AMPs undergo complex post-translational modifications, such as glycosylation. As a result, in some circumstances, the chemical synthesis of AMPs is considered inefficient for mass-scale production [45]. Large-scale AMP manufacturing is now possible thanks to recent advancements in recombinant DNA technology. Foreign genes can be cloned into specialized vectors and expressed in prokaryotic or eukaryotic host cells using this technique. In terms of time and production costs, this has been determined to be the most efficient method [48].

To generate heterologous peptides of different lengths, folds, and complexities, a variety of expression systems are used. When choosing a host system for their creation, the length of protein and peptides, intracellular localization, proper bending, and glycosylation pattern are all key factors to consider [49]. Bacteria and yeasts are the principal hosts for AMP production, accounting for 97.4% of heterologously produced AMPs, as shown in a recent study [50]. Plants have recently been identified as potential hosts for AMP production. In the following sections, each organism will be discussed in-depth in terms of its use as an expression platform.

### 2.1. Bacterial Expression System

The most frequently applied microorganism for AMP production is the bacterium *E. coli* [46]. *E. coli* has been shown to be the most cost-effective candidate for recombinant protein production due to its rapid proliferation, wide accessibility, well-established DNA manipulation processes, and comprehensive understanding of its genetics [51]. However, some hurdles must be overcome before AMP production in *E. coli* can be successful. Preventing the AMP’s natural activity is the first step in avoiding lethality to the host strain. The second step is to overcome the AMP’s instability due to its synthetic characteristics and size [52]. As a result, the AMP is commonly linked to a carrier protein with anionic properties to ensure effective expression.

The Gram-positive bacterium *B. subtilis*, in contrast to the well-known Gram-negative bacterium *E. coli*, is thought to be a safe organism [53]. As a result, screening resistance genes in *B. subtilis* rather than *E. coli* is preferable. Two fundamental differences distinguish the efficacy of antimicrobial gene screening in *B. subtilis* and *E. coli* expression systems. The first distinction is the host cell; *B. subtilis* has a natural potential to release proteins into their surroundings, often at high concentrations [54], which reduces host cell toxicity, whereas *E. coli* secretes proteins into its periplasmic space, resulting in a toxic state in the host cells. The expression vectors used in the *B. subtilis* and *E. coli* systems differ as well, with the *B. subtilis* system using the constitutive expression vector pBE-S and the *E. coli* system using the inducible expression vector pET-22b [38]. Despite the *E. coli* system’s obvious drawbacks, the use of high-efficiency *Bacillus* secretion has been restricted to large quantities of industrial enzyme production. This approach has several drawbacks, including the difficulty of producing some mammalian proteins, protein buildup as inclusion bodies, and protease contamination from host proteins [53]. These limitations have been thoroughly studied for several decades, and we will demonstrate some of the strategies that have been proposed by addressing recent efforts to express potential proteins in the *Bacillus* expression system.

### 2.2. Yeast Expression System

Yeasts like *Saccharomyces cerevisiae* and *Pichia pastoris* are often employed as expression systems. It takes advantage of both bacterial and eukaryotic expression systems [47]. This system is responsible for both secretory and intracellular protein expression. The majority of recombinant proteins manufactured in recent years have been featured by *S. cerevisiae* [55]. Because of the significant investigation into its genetics, this species is generally recognized as a host for heterologous protein production. Meanwhile, the numerous studies that have used *P. pastoris* as a host for heterologous expression have grown dramatically in recent years [56,57]. Because *P. pastoris* is not a fermentative microbe like *S. cerevisiae*, it can be cultured at much higher concentrations without producing hazardous byproducts. Other benefits of using *P. pastoris* include increased levels of heterologous gene expression, convenient scale-up, low-cost growth media, simple purification, and the ability to perform post-translational modifications [58]. Protein hyperglycosylation is one of the key disadvantages; unlike mammalian systems, it provides both N and O-linked oligosaccharides on proteins; and high protein output requires fermentation.

### 2.3. Plant Expression System

Plants that have been genetically modified to allow for heterologous expression of proteins are being established, primarily for crop development. As a result, no attempts have been made to quantify the amount of recombinant AMP generated [47]. Furthermore, the amount of peptide produced is heavily focused on promoter selection, non-target genomic insertion, transgene copy number, and target tissue [59]. Plant expression systems, on the other hand, have been created as a viable option for creating peptides that do not require regulated expression, specialized folding, or processing mechanisms. However, plants display some challenges that must be overcome. For example, adding highly immunogenic plant-specific sugars to N-linked glycans is a significant barrier that must be overcome to create proteins in plants [47]. Tobacco was used in another initiative to humanize heterologous protein synthesis by expressing the mammalian specific (1,4)-galactosyltransferase [60]. This knowledge, however, has not yet been applied to the large-scale production of genetically modified plants. Taken all together, plant expression systems represent an extremely promising field for future research. Table 1 shows a list of AMPs isolated from various expression systems, along with their respective roles.

## 3. CDNA Library Based Plant AMPs Isolation

In functional cloning, cDNA libraries are commonly utilized to identify genes based on the function of the encoded protein. Furthermore, because cDNA lacks introns, it can be expressed in prokaryotic cells. To accomplish this, cDNA libraries are generated using a reverse primer depending on the mRNA poly-A tail and forward degenerate primers relying on heavily conserved signal regions of the peptide precursors [91]. These are used to selectively amplify the target AMP cDNAs. As a result, this approach identifies inadequacies, discusses the most probable origin, and suggests ways to feasibly resolve them. However, several cDNA library techniques have been studied to clone AMPs in recent years [92,93,94] because of their positive effects, such as correctly transcribed genes and cloning ability [95]. Moreover, the absence of introns allows engineering via a bacterial expression system. Following this, *B. subtilis* has been identified as an ideal organism for studying the increased expression of foreign proteins [96]. It has long been used successfully to express many therapeutic molecules, such as some industrial enzymes, e.g., lipases, proteases, and amylases, because of its comparatively simple structure of the cell, rapid growth, short fermentation duration, and strong ability to disperse proteins immediately into the outer membrane [97,98]. Recently, the Key Lab of Crop Disease Monitoring and Safety Control in Hubei Province, Huazhong Agricultural University, Wuhan, Hubei, China, proposed a new method to isolate resistance genes by using the *B. subtilis* expression system, which showed a significant potential against the superbug bacterial and fungal plant pathogens [36,39,70]. The details of this approach are discussed below.

### 3.1. CDNA Library Construction

This approach aims to build a plant cDNA library in the *B. subtilis* expression system and then search for the possible antimicrobial gene. According to this approach, the candidate plant seeds are grown in nutrient soil pots in a growth chamber at 28 °C under dark conditions. After three weeks of growth, the candidate plant leaves are inoculated with 48-hour-old PDA disks of a *Rhizoctonia solani* WH1. The leaves are sampled 9 times after inoculation: 0 to 96 h, collected every 12 h, and then immediately frozen in liquid nitrogen and stored in a -80 °C freezer. The development of candidate plant cDNA libraries is carried out in accordance with standard procedures. The TRIzol reagents are used to extract the total mRNA. Then, the mRNA is purified with the PolyATtractR mRNA isolation system (Promega, 2800 Woods Hollow Road, Madison, WI 53711, USA). Constructing a cDNA library with a cDNA kit (Takara, Dalian, China, Biomedical Technology) with unique oligo dT primers (containing an *Xba* I cleavage site) and integration of cDNA with three pairs of primers containing a *Nde* I cleavage site. After that, the cDNA and the pBE-S vector are digested with *Xba* I and *Nde* I and then ligated with T4 ligase under the following PCR conditions: 16 °C for 11 h, 65 °C for 30 min, and 12 °C for hold. To spread more vectors, the ligation mixture is placed into a competent *E. coli* HST08 cell. Using the EasyPure^®^ Plasmid MiniPrep Kit (TransGen, No. 1 North Yongtaizhuang Road, Haidian District, Beijing-100192, China), *E. coli* cell plasmids are isolated and then transformed into *B. subtilis* SCK6 cells [36,38]. The pBE-S vector containing a subtilisin promotor (aprE-P) and a peptide secretion signal (aprE-SP) are used in this proposed method. The signal secretion peptide is extracted from *B. subtilis,* which is located upstream of the multiple cloning sites (MCS) [99]. The sequence of His-tag is positioned downstream of the MCS, enabling target proteins to scan for successful secretory signal peptides. Then, 96 monoclonal clones are randomly chosen from *B. subtilis* for PCR analysis to verify the accuracy of the cDNA library under the following criteria: 95 °C for 5 min, 95 °C for 30 s, 55 °C for 30 s, 72 °C for 1 min (28 cycles), and 72 °C for 5 min. To estimate the validity of cDNA library colonies, the primers pBE-S-F (5’-GTTATTTCGAGTCTCTACGG-3’) and pBE-S-R (5’-TAACCAAGCCTATGCCCCTACA-3’) were used.

### 3.2. CDNA Sequencing Analysis

**Figure 1:**
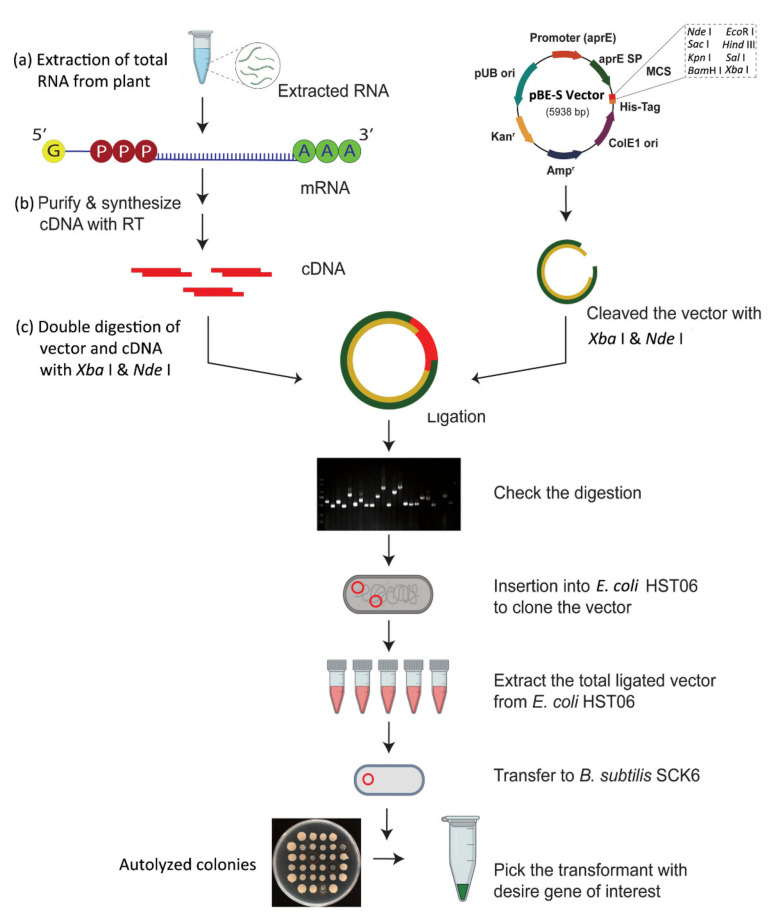
Overview of the cDNA library construction procedure through the *B. subtilis* expression system. (a) Extraction of total RNA from the plant and (b) synthesis of cDNA strand with reverse transcription (RT) process. (c) After that, selection of pBE-S vector and synthesis cDNA digested with *Xba* I and *Nde* I, and then ligation with T4 ligase. Finally, transfer of the modified vector into the *B. subtilis* expression system.

cDNA library quality is evaluated using gel electrophoresis and sequence analysis. The clones of the cDNA libraries are placed in the refrigerator at −80 °C [39]. The overall procedure of cDNA library construction is presented in Figure 1. The primers for constructing the cDNA library can be found in Supplementary Table S1. The PCR product is then sent for Sanger sequencing to determine the cDNA sequences. The Basic Local Alignment Search Tool (BLAST) is used on NCBI (https://blast.ncbi.nlm.nih.gov/Blast.cgi/; accessed date: 3 January 2020) to validate the sequences of cDNA library data and their location on a chromosome of the desired plant genome database. A translation tool (https://web.expasy.org/translate/; accessed date: 4 January 2020) is also used to translate the cDNA sequences into amino acid sequences based on the codon of each amino acid (according to the NCBI codes). However, the screening principle suggests that expressing a potential resistance gene (in a *B. subtilis* cell) causes cell autolysis, and by analyzing this occurrence, the target antimicrobial genes are obtained.

### 3.3. Bioinformatics Analysis of Candidate Peptides

AMPs are distinct biomolecules that are classified into subsets based on amino acid content and configuration and are crucial for peptide optimization and modification. These peptides’ secondary structures are estimated using computational methods and classified into four classes based on their structure-function relationship: (1) α-helices, (2) β-strands, (3) both α-helix and β-strand, or (4) extended (non-αβ) [38]. There is a wide range of software and online servers that can be used to estimate the structural configuration of peptides and reveal their functional properties. The overall bioinformatics analysis is briefly described below.

#### 3.3.1. In Silico Prediction Analysis 

The identified sequences of amino acids were uploaded to AMP prediction servers in FASTA format. For overall prediction, two servers were used: the Collection of Antimicrobial Peptides (CAMP_R3_) [100] and the Database of Antimicrobial Peptides (ADAM) [10]. AMP prediction servers such as iAMPpred (http://cabgrid.res.in:8080/amppred/server.php/; accessed date: 4 January 2020) [101] and AntiBP (https://webs.iiitd.edu.in/raghava/antibp/index.html; accessed date: 4 January 2020) [102] are used to reveal the spectrum of predicted AMP antimicrobial properties based on pathogen type.

#### 3.3.2. Prediction of Physiochemical Properties of AMPs

To predict the physiochemical properties of candidate peptides, different servers can be used. The antimicrobial peptide database (APD3) server (https://wangapd3.com/main.php; accessed date: 5 January 2020) predicts the amino acid composition, molecular weight, net charge, total hydrophobic ratio, and Boman index [103], whereas the DBAASP server (https://dbaasp.org/; accessed date: 5 January 2020) predicts the isoelectric point and in vitro accumulation of AMPs [104]. HeliQuest websites [105] (https://heliquest.ipmc.cnrs.fr/; accessed date: 5 January 2020) can be used to evaluate peptide helix forms (amino acids sequence). Besides, the PSSpred database (https://bio.tools/psspred; accessed date: 5 January 2020) predicts the secondary structure of AMPs [106].

#### 3.3.3. 3D Structure Prediction

For 3D structure prediction, the HeliQuest online tools can be used to generate the helix wheel graph. The I-TASSER [107] and PEPFOLD 3 [108] predict the 3D structure of the candidate peptide, which is then visualized using the UCSF Chimera 1.14rc software system. To validate the peptide’s 3D structure, the PROSAII [109] and MolProbity web tools [110] can be used. 

Despite the relatively simple cloning and production of AMPs in vitro, the vast diversity of AMPs’ structure affects their antimicrobial activity and mechanisms of action. Understanding the peptide structure through in silico studies is crucial for overcoming these consequences. In our lab, we observed that different structures could result in a broad spectrum of biological activities. According to bioinformatics predictions, the majority of our isolated antimicrobial peptides contain a significant proportion of hydrophobic cationic residues, which promote connection with the fatty acyl chains and enhance specificity for negatively charged cell membranes of pathogens over zwitterionic mammalian membranes. As a result, despite the α-helices peptides, several antimicrobial peptides containing both α-helices and β-strands demonstrated strong antibacterial activity.

### 3.4. Candidate Protein Extraction from the B. subtilis Expression System

Using the ammonium sulfate precipitation process, extracellular peptides are precipitated [70]. The strains, stored in the −80 °C refrigerator, were removed and streaked on kanamycin-containing LB plates and cultured for 12 h at 37 °C. Single colonies grown on kanamycin-containing plates were selected and shaken at 180 rpm for 60 h in LB. In a 50 mL centrifuge tube, 15 mL of the culture solution is centrifuged at 4 °C, 10,000 rpm for 25 min. In a pre-cooled beaker, the supernatant was filled and placed on ice, and then a saturated ammonium sulfate solution was continuously applied to the beaker while the liquid was stirred until the solution was cloudy. The beaker was placed overnight in a 4 °C refrigerator to precipitate the protein, and the fluffy protein was aspirated in a 50 mL centrifuge tube and centrifuged at 4 °C for 25 min, at 10,000 rpm. The supernatant was decanted with 1 mL of pre-cooled PBS solution, and the protein was dissolved. A spectrophotometer is used to measure the concentrations of the peptides. Precipitated peptides are immersed in a buffer of 25 mM PBS (pH 7.0) and dialyzed for 24 h at 4 °C in the same PBS buffer. To discard the insoluble debris, centrifugation is used. Then these extracted recombinant proteins are sent for the purification steps.

### 3.5. His-Tag Fusion Peptide Purification

The hexahistidine tags (His-tags) are one of the most widely used tags for recombinant protein affinity purification [111]. The benefits of these tags over other recombinant protein purification are: (1) the tag can be incorporated into a protein C- and N-terminus, (2) the tags have lower protein structure interactions due to their limited length and neutral charge at physiological pH values, and (3) the proteins can be dissolved after affinity chromatography under relatively mild conditions [112]. 

#### 3.5.1. Immobilized Metal Affinity Chromatography

His-tagged proteins can be purified by single-step affinity chromatography, namely immobilized metal ion affinity chromatography (IMAC), which is typically present in several kinds of configurations. The most widely used for separating protein/peptides are Ni-NTA matrices [113]. The His-tag residues interact easily with transition metal ions such as Ni^2+^ immobilized on beads or a resin for purification because of their high affinity for the metal ions and bind tightly to the IMAC column. IMAC is a general mechanism of affinity based on the binding of the His-adjacent tags histidine to an immobilized divalent metal ion. The list of certain specific fusion tags facilitating the affinity chromatography is presented in Table 2.

**Figure 2:**
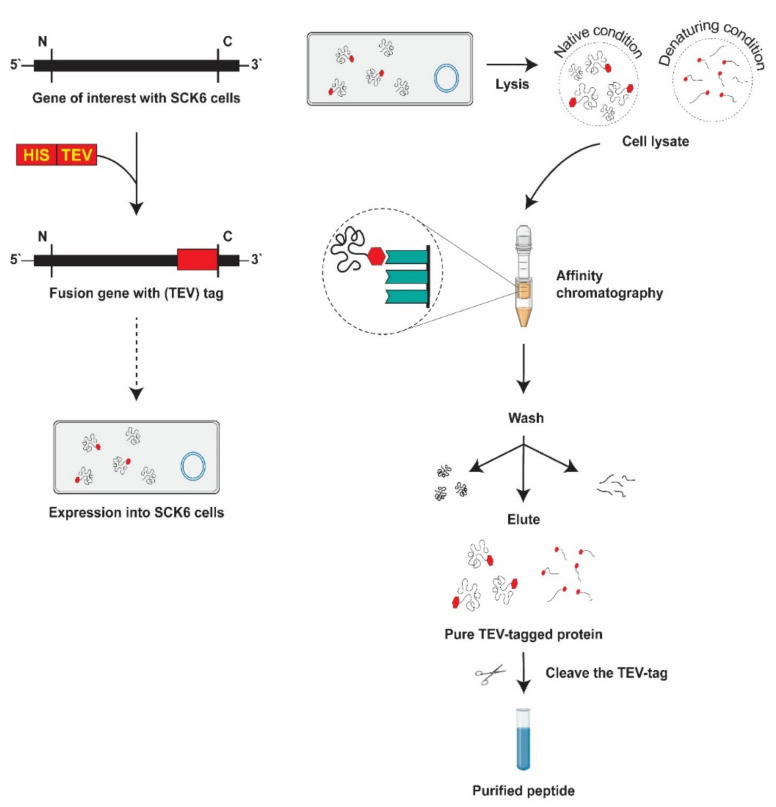
Scheme of procedures used in the purification of antimicrobial peptides by 6xHis tag purification method. A specific tag is initially fused to the target peptide at the DNA level to express tagged fusion protein so that the peptide solution is retrieved and added to a column containing specific relevant protein ligands. Washed undesirable compounds and finally, a TEV-protease to cleave the target peptide from the affinity tag.

For Ni-NTA purification, a cleared lysate from *B. subtilis* cell pellet is obtained for His-tagged proteins purification using IMAC resin. In this procedure, interaction is performed in a batch system because it is the most accurate approach, particularly when the target protein is only detectable in small quantities. First, we gently inverted the bottle several times to suspend the IMAC resin, then transferred 1 mL to a 15-mL conical centrifuge tube. The resin was delivered as a 50% slurry, equivalent to a 0.5 mL bed volume. This allows the natural settlement before removing the supernatant. To adjust the resin, we added 2.5 mL of basis buffer (50 mM NaH_2_PO_4_, 300 mM NaCl, 10 mM Imidazole, pH-8) and shook it thoroughly. We allowed the resin to settle again by gravity, then removed 2 mL of the supernatant. Then, we added 10 mL of previously prepared cleared cell lysate to the equilibrated IMAC resin and incubated at 4 °C for 25 min. We filled the binding suspension into a reusable gravity flow column with a capped bottom outlet, removed the bottom cap of the column, and collected the flow-through. The column was washed with 5 mL of wash buffer (50 mM NaH_2_PO_4_, 300 mM NaCl, 20 mM Imidazole, pH-8). Using 0.5 mL elution buffer (50 mM NaH_2_PO_4_, 300 mM NaCl, 500 mM Imidazole, pH-8), elute the His-tagged protein five times [126]. We collected each eluate in a separate tube and calculated the protein concentration of each fraction. If it is not used for a long time, it can be sealed by adding about 4 mL of 20% (sterilized) ethanol and stored at 4 °C. The process is simple, cheap, and straightforward, which has driven extensive usage. The overall purification process is illustrated in Figure 2.

#### 3.5.2. Elimination of Tag

There are several potential advantages of using fusion tags for the processing and purification of recombinant proteins, but they interfere with the final use of the protein; therefore, they need to be removed. Some plasmids are intended to add a protease recognition site between an inserted tag and the protein of interest. The proteases and their recognition sites that are widely used are given in Table 3. In our laboratory, we use TEV (Tobacco Etch Virus) proteases to remove recombinant proteins from connected fusion partners because of their rigorous sequence accuracy and activity over a broad thermal spectrum. In general, TEV protease is a unique cysteine protease that identifies the Glu-Asn-Leu-Tyr-Phe-Gln-(Gly/Ser) amino acid sequence and cleaves between the residues of Gln and Gly/Ser. This enzyme is widely used to eliminate the fusion protein His-tags. After the label is digested, there is only one additional Gly/Ser amino acid residue at the N end of the target protein. Hence, reducing the impact on the structure and activity of the target protein and creating clear peptides. However, the His-tag is tiny (6–10 amino acids), and the recombinant protein does not always need to be extracted before use. In reality, the study stated that the tags had no significant impact on the structure of the linked protein [127]. 

### 3.6. Tris-Tricine SDS-PAGE and Western Blotting

To estimate the target peptides’ molecular weight, they were separated using SDS-PAGE and electrophoresis with a Tris-Tricine buffer system. The isolated protein was transferred via a pre-stained marker to the polyvinylidene difluoride (PVDF) membrane at 21 V and 185 mA for 21 min. PVDF membranes are blocked for 2.5 h in 2.5% nonfat dry milk, transferred overnight to a nonfat dry milk mouse containing primary mouse antibody (1:10,000, Frdbio). Then the PVDF was washed three times with 0.1% Tween-20 (PBST) in phosphate-buffered saline for 10–15 min. The chemiluminescence (ECL) western blot assays are used to detect the bands after incubation with peroxidase-conjugated goat anti-mouse antibody IgG (H + L) for 3 h followed by a three-time wash with PBST [39].

## 4. Characterization of AMPs

**Figure 3:**
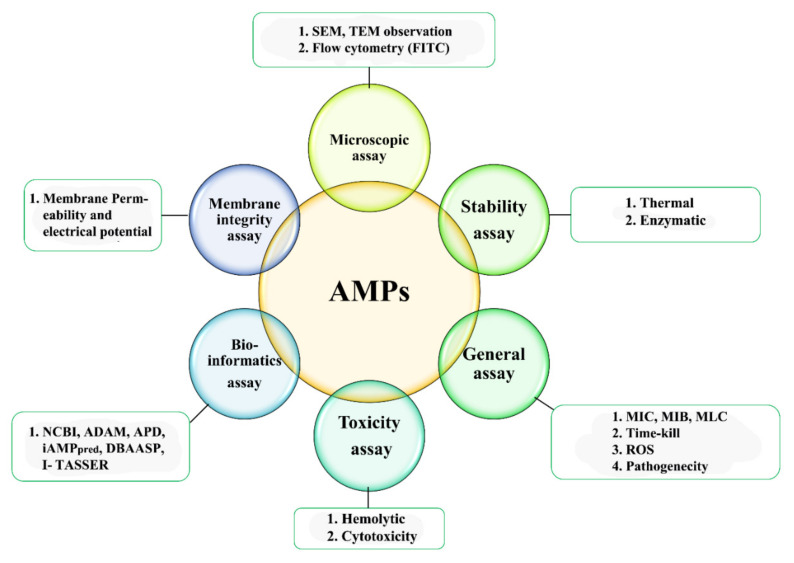
Different techniques for AMPs characterization.

The AMP characterization provides an overall understanding of the structure, efficacy, and biochemical characteristics of a peptide that is very important to choose as a drug candidate. The technique of characterization, however, can be divided into two categories: qualitative techniques based on morphological makeup and physical characteristics and quantitative methods relying on chromatographic structure and quantified results. Furthermore, a non-fatal, quick, fast, and smaller scale of sample involving procedures of characterization is preferable. It is necessary to consider that when used alone, none of the proposed approaches are adequate to describe the AMPs well. Therefore, two or more methods are generally used which support one another (Figure 3).

### 4.1. Microscopic Studies 

AMPs can effectively incorporate into bacterial membranes due to their amphiphilic properties, which is their primary antimicrobial mechanism. To determine how the novel peptides affect the outer layers of bacteria, we investigated the microscopic alterations that occur in pathogenic organisms as a result of treatment with AMPs under various conditions. SEM is used to visualize host cells after they had been treated with different concentrations of AMPs for different time intervals (12, 24, 48, and 72 h, respectively). On the other hand, the TEM observation step is similar to the SEM [72]. However, after incubation, the bacterial cells were collected and adjusted to a glutaraldehyde solution of 3% (*v/v*). A transmission electron microscope was then used to examine the samples.

### 4.2. Hemolytic Activity 

Antimicrobial peptides must be nontoxic to erythrocytes to be useful in widespread applications. This is usually determined by the AMPs’ capacity to denature mammalian red blood cells. For this experiment, sheep [143], rats [144], pigs [145], and rabbits red blood cells [146] were considered. There is only a limited report that compares the hemolytic behavior of various peptides against red blood cells of different species of mammals [145,147]. These suggest that the hemolytic activity heavily relies on the source of a red blood cell. However, in our laboratory, we used sheep blood for conducting this investigation [39,70]. As a positive control, a 1 percent Triton X-100 treated erythrocyte suspension is used, whereas a solution incubated with just a PBS buffer is used as a negative control. The following equation determined the percentage of hemolytic activity of the peptide: hemolysis (percent) = [(OD_540_ peptides − OD_540_ buffer)/(OD_540_ Triton X-100−OD_540_ buffer)] × 100%. 

### 4.3. Minimum Inhibitory Concentration (MIC) 

MIC is a test that defines the minimal concentration of antibacterial agents required to inhibit the indicator organisms’ variable growth. A suspension of the indicator organism is made at a concentration of one million colony-forming units (CFU) per ml. The peptide is then mixed and incubated with the indicator of the bacterial organism to various concentrations. Using different concentrations of the peptides in a 96-well flat-bottom Microtiter^®^ plate, MIC is calculated for liquid growth inhibition assays by standard procedures (CLSI) [148]. In two separate study sessions, both triplicate trials are performed, and for each number of samples, both positive (no peptide) and negative (no bacteria) controls can be used. 

### 4.4. AMPs Stability Determination

To check the strength of the AMPs against various temperatures and enzymes, stability tests are carried out. For thermal stability screening in our laboratory, the peptide is heated at various temperatures (4 °C, 50 °C, 70 °C, and 100 °C) for 30 min before being tested for use. Meanwhile, different enzymes (trypsin, lipase, pepsin and amylase at 30 °C, protease E, and peptidase K at 57 °C for 1 h) are used to test the sensitivity of peptides. The agar diffusion test is performed to characterize the stability of peptides on different Gram-positive and Gram-negative bacteria [36,39,70]. Importantly, we have verified that pure antimicrobial peptides are stable and resistant to enzymes at higher temperatures.

### 4.5. Cell Membrane Integrity

Propidium iodide (PI) staining is used to further investigate the impacts of our AMPs on cell membrane integrity. PI is a nucleic acid dye that penetrates the cell through a damaged membrane and stains when it binds to a double-stranded nucleic acid. Confocal microscopy and flow cytometry [38,149] can be used to investigate the membrane integrity of the cells loaded with antimicrobial peptides. In the logarithmic growth step, the bacterial cells are grown, and the concentration is set to OD_600_ (0.1–0.2) and incubated for 2 h at 28 °C with antimicrobial peptides. Images of fluorescence intensity were obtained FACSVerse system (BD, Franklin Lakes, NJ, USA), and CyExpert 2.4 software was used to interpret the flow cytometry data. The wavelengths of the excitation and emission in this assay are 535 and 617 nm, respectively.

### 4.6. Reactive Oxygen Species

Antimicrobial peptides may cause a microbial reactive oxygen burst in the method of impeding pathogenic microbes, which is widely recognized as one of the strategies of antimicrobial peptides. The 2,7 dichlorodihydrofluorescein (DCFH) generated by the hydrolysis of 2,7dichlorodihydrofluorescein diacetate (DCFHD-DA) in cells can be oxidized by reactive oxygen species to produce green fluorescence. The fluorescence intensity of the resuspended cells is measured using an ELISA (SPARK) microplate reader (excitation 485 nm and emission 540 nm), and images are captured using a fluorescence microscope [39].

## 5. Mode of Action of AMPs

In the last couple of years, the study on AMPs mode of mechanism increased significantly [150]. Heterologous functions of the peptides encourage knowing the mechanism. The potency of AMP depends on several factors, e.g., peptide concentration, microbial growth phase, membrane permeability, and composition [151]. Although the action mechanisms are reviewed in several studies, none of the previous work collectively summarizes the comprehensive mechanism of action of AMPs against different pathogens, including bacteria, fungi, and viruses. Membrane permeabilization of AMP plays a vital role in exerting its action. Plant-AMPs are most often rich in cysteine and offer greater chemical and proteolytic stability [152]. At the early stage, the mode of action was thought to only have electrostatic interactions between cationic peptides and negatively charged cell surface proteins. However, studies on anionic peptides revealed that they were also able to interact with the cell membrane by adopting α-helixes and β-sheets [153]. The non-specific region targeting ability largely depends on the peptide’s vibrant confirmation, with transitional stages taking place earlier or throughout the binding. To describe the interaction between AMPs and the cell membrane, four mechanistic models have been proposed: barrel-stave pore model, carpet model, toroidal pore model, and disordered/detergent toroidal pore model.

### 5.1. Barrel-Stave Model

**Figure 4:**
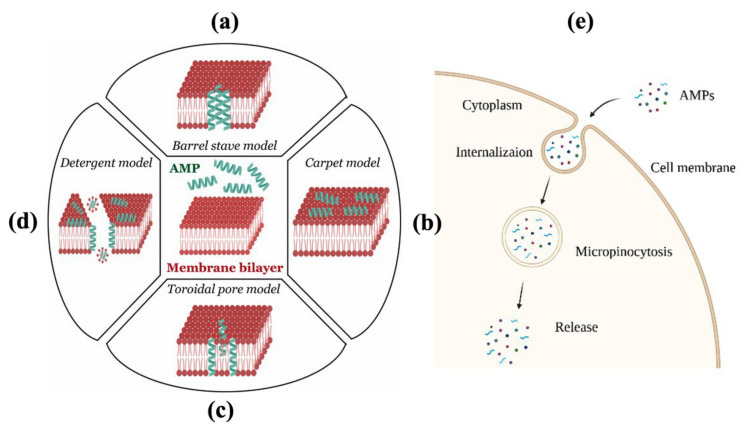
Energy independent and dependent mode of action of plant-derived AMPs. The energy-independent mechanism follows four different models (**a**–**d**), and the energy-dependent mechanism follows micropinocytosis (**e**) to release AMPs in the cytosol.

According to the barrel-stave model, cationic AMPs with α-helical structures internalize through the membrane via transmembrane pores. However, peptide assembly must take place earlier than internalization [154,155]. The hydrophobic moiety of the peptide aligns parallel to the lipid bilayer of the membrane, and the hydrophilic moiety of the peptide points inward to make the pores. This arrangement allows the formation of transmembrane pores (Figure 4a). After being internalized, the peptides enter into the cytosol and hence start interacting with intercellular components. 

### 5.2. Carpet Model

This is commonly known as the membrane solubilization mechanism. This phenomenon does not require the involvement of peptide internalization. Diffusion of peptides across the membrane leads to peptide aggregation and membrane disruption (Figure 4b). Initially, the peptides bind onto the bilayer membrane surface and cover it like a carpet. This causes lessening of the bilayer homogeneity and resistance. Later, they aggregate with each other and destabilize the membrane [156]. The advantage of this model is that the peptide-lipid interaction does not require any specificity. For example, wheat-derived type-1 thionins (α1-, α2-, β-purothionin) and plant defensins (AtPDFL2.1, ZmD32, PpDFN1, PsDef1) undergo cell penetration via the carpet mechanism [157,158].

### 5.3. Toroidal Pore Model 

The toroidal pore model cannot be described fully by either the Barrel-stave or the carpet model. Therefore, it is known as an intermediate model. After being absorbed on a membrane surface, peptides will be aggregated and induce bending of the bilayer. The hydrophobic part of the membrane contacts the lipid membrane layer, while the hydrophilic part remains inside [159]. Hence, the appearance of toroidal pores allows peptide internalization through the destabilized membrane (Figure 4c). 

### 5.4. Disordered/Detergent Toroidal Pore Model

This is more likely a random way to create pores and destabilize lipid membranes [160]. The lipid bilayer tends to twist inward, and the peptides stabilize the bent membrane (Figure 4d). The deeply integrated peptides stabilize flat, curved pores, while the residual peptides are rearranged and stabilize the membrane’s bend. Due to the catastrophic collapse of the membrane, it cannot retain the contents and results in cell death. 

Another common mechanism followed by AMPs is micropinocytosis. It is an ATP-dependent pathway, where the lipid bilayer folds inside, has contact with peptides, and forms vesicles. These so-called macropinosomes allow the internalization of AMPs and subsequently exert antimicrobial action effectively [161]. Endocytosis may categorize into phagocytosis (specific uptake) and micropinocytosis (non-specific uptake). During micropinocytosis, the lipid bilayer membrane folds inside and forms vesicles called macropinosomes. AMPs are trapped inside the macropinosomes that resemble membrane structure and allow internalization in the cell (Figure 4e).

Plant-derived AMPs can target cell membrane lipids or cell wall composition [162]. For example, thionins are expressed in seeds and leaves of both monocots and dicots that are toxic to microbes [9]. Moreover, defensins have potential antifungal activity due to their specific interaction with sphingolipids (a major membrane component in the fungi) [163]. Additionally, this interaction triggers downstream signaling cascades and stimulates programmed cell death of fungi [62]. Antiviral peptides target the viral envelope or host cell membrane and cause neutralization or membrane instability. For example, plant defensins are one group of the most studied AMPs that exert inhibition of viruses [164].

**Figure 5:**
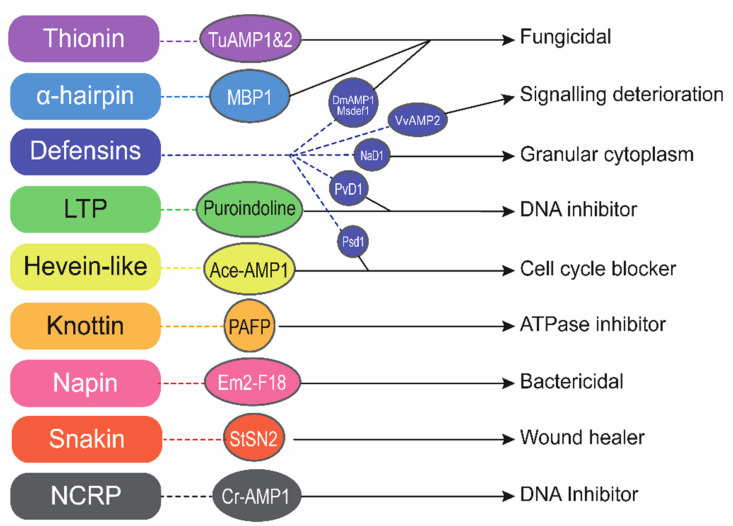
Cellular targets and the associated role of different plant-derived AMPs. Herein, different classes of AMPs (left) are connected with representative examples by dotted colored lines. Functions of the AMPs (right) are connected with the cellular targets by solid black lines. Abbreviations: lipid transfer proteins, LTP; non-cysteine-rich-peptides, NCRP; antimicrobial peptide 1 & 2 of *Tulipa gesneriana*,TuAMP1&2; myelin basic protein 1, MBP1; antimicrobial peptide 1 from *Allium cepa*, Ace-AMP1; anti-fungal protein of *Phytolacca Americana*, PAFP; peptide from *Eugenia malaccensis*, Em2-F18; snakin-2 from *Solanum tuberosum*, stSN2; cathelin-related antimicrobial peptide, cr-AMP1; antimicrobial peptide 1 of *Dahlia merckii*, DmAMP1; antimicrobial peptide 2 of *Vitis vinifera*, VvAMP2; *Nicotiana alata* defensin 1, NaD1; *Phaseolus vulgaris* defensin 1, PvD1; *Pisum sativum* defensin 1, Psd1. The concept of the figure was taken from Goyal and Mattoo, 2016 [167].

The mechanism of plant AMP-mediated cellular damage is still unknown as the cellular targets by AMPs are constantly changing. Recent work suggests that the membrane is not the only target of plant-derived AMPs as bacteria can survive even after the membrane is damaged. Hence, other targets of AMPs that direct microbes to death include inhibition of DNA synthesis, protein synthesis, protein folding, mitochondria, and other intracellular targets [165,166]. Most often, the degree of permeabilization does not correlate with the activity. Studies revealed that the action of AMPs is associated with proteins/enzymes, nucleic acids, and other intracellular targets [165]. After being internalized, AMPs target cellular organelles and interrupt their regular functions. These plant-derived peptides follow the miscellaneous mode of action. Hence, they can strike a large number of targets. Goyal and Mattoo reviewed detailed facts and targets of plant-AMPs [167]. Some of the major plant-AMPs and their associated targets are illustrated in Figure 5.

## 6. Conclusions

We live in a world populated by these green species, even though plants are continually subjected to many diseases and pests. This finding shows that plants have developed remarkably impactful defensive skills that are used to impede tissue-damaging invaders’ growth. Recently, significant advances in the decoding of the plant immune system’s genetic and molecular basis have helped researchers envision a theoretical model of how the plant perceives a challenging environment produced by a microbial attack and eventually converts it into an ideal defense mechanism. In this sense, antimicrobial peptides (AMPs) with various functional and antimicrobial characteristics are promising candidates for pathogen control, including drug-resistant microbes. The ubiquitous existence of these tiny compounds among plant species is demonstrated by the fact that they offer quick, effective, and long-lasting immunity against a wide variety of pathogens. In fact, because of their importance in proving that plants thrive in natural environments and their tremendous skill in agronomy and pharmacy, plant AMPs have now become an intensively studied subject. It is also possible to isolate AMPs from plant sources in different ways and to adjust the testing procedures to the processing of such peptides or members of peptide groups due to their huge potentiality in bioprospecting. In this review, we introduced a new, responsive and elevated approach to isolate genes for antimicrobials. In sum, this approach integrates a library of cDNA into the expression system of *B. subtilis*. It allows thousands of expressed proteins to function from within the cells of *B. subtilis*. By analyzing cDNA libraries and testing autolyzed clones, it is feasible to identify resistance genes rapidly. These target genes are expressed by *B. subtilis*, which serves to acquire the secreted proteins for pathogen defense as an ideal expression system. Furthermore, we revealed the usefulness of AMPs as a defensive mechanism by their widespread distribution in the plant family and impressive diversity in the composition of the host peptide. Moreover, the ongoing production and manufacture of novel AMPs with potential biological features derived from plants should offer excellent prospects for further expanding their use in treating diseases of animals, humans, and plants.

## Figures and Tables

**Table 1:** List of AMPs/recombinant proteins produced by different expression systems.

Expression System-AMPs/Recombinant Protein	Source	Specific Role	References
***Escherichia coli***			
Thaumatin-like protein (ATLP3)	*Arabidopsis thaliana*	Fungal growth inhibition	[61]
Osmotin-like protein (SnOLP)	*Solanum nigrum*	Mycelium growth inhibition (*Phytophthora nicotiana, Fusarium solani, Colletotrichum gossypii*)	[62]
Mi AMP1	*Macadamia integrifolia*	Spore and mycelium growth inhibition	[63]
Pg-AMP1	*Guava psidium*	Gram-positive (*Staphylococcus* sp.) and Gram-negative (*Pseudomonas* sp.) bacterial growth inhibition	[64]
Vv-AMP1	*Plant Vitis vinifera*	Fungal growth inhibition	[65]
Puroindoline A	Wheat seed	*Staphylococcus epidermidis* growth inhibition	[66]
RHG1-LRR	*Glycine max*	Fungal mycelium growth reduction	[67]
Transglutaminase (TGZ)	*Zea mays*	Resistance to phytopathogens	[68]
***Bacillus subtilis***			
Thionin	*Oat*	Inhibition of bacterial infection	[69]
β-purothionin	*Z. mays*	Controlling bacterial and fungal infection	[48]
AsR416	*Allium sativum*	Bacterial infection reduction	[38]
AsR498	*A. sativum*	Bacterial infection reduction	[38]
IiR515	*Isatis indigo*	Bacterial and fungal growth inhibition	[70]
liR915	*I. indigo*	Bacterial and fungal growth inhibition	[70]
CeCPI	*Colocasia esculenta*	Suppression of gall formation in tomato	[71]
AtR100	*A. tauschii*	Bacterial (*Clavibacter fangii., Clavibacter michigenesis*) and fungal (*B. cinerea*) infection reduction	[36]
AtR472	*Aegilops tauschii*	Bacterial (*C. fangii., C. michigenesis*) and fungal (*B. cinerea*) infection reduction	[36]
ZM-985	*Z. mays*	Bacterial and fungal growth inhibition	[72]
ZM-804	*Z. mays*	Bacterial and fungal growth inhibition	[37]
OrR214	*Oryza rufipogon Griff*	Resistant to bacterial and fungal infection	[39]
***Saccharomyces cerevisiae***			
AtPTR1	*A. thaliana*	Reduced phytopathogens infection	[73]
*A*tChx17	*A. thaliana*	Transcriptional inhibitor	[74]
HKT1	*Tritichum aestivum*	Salt and stress-tolerant	[75]
AtGAT1	*A. thaliana*	Plasma membrane integrating role	[76]
AtINT2	*A. thaliana*	Encoded truncated protein to inhibit transmembrane helices	[77]
***Pichia pastoris***			
Antifungal Protein (AFP)	*Aspergillus*	Antifungal activity	[78]
Defensin (Pdc1)	Corn	Fungal mycelium and conidial growth reduction	[79]
Defensin (Psd1)	*Pisum sativum*	Resistant to fungal infection	[80]
LCT1	*Tritichum aestivum*	Cationic promoter	[81]
***Baculovirus*-mediated insect cell**			
Patatin	*Solanum tuberosum*	Exhibit enzymatic activity	[82]
Cyclin-dependent kinase A (CDKA)	*A. thaliana*	Control cell cycle activation	[83]
Acyl-CoA synthetase	*A. thaliana*	Provides energy during germination	[84]
Ethylene-inducing xylanase	*Nicotiana tabacum*	Not clear	[85]
**Plants system**			
AP24 osmotine	*N. tabacum*	Resistance to *Phytophthora infestens, Rhizoctonia solani, Fusarium solani*	[86]
RsAFP2	*Raphanus sativus*	*Resistance to Fusarium graminearum* and *Rhizoctonia cerealis*	[87]
Defensin 1 (BrD1)	*Brassica rapa*	Resistance to *Nilaparvata lugens*	[88]
NmDEF02	*N. tabacum*	Enhanced crop improvement	[89]
MsDef1	*Medicago sativa*	Resistance to *Fusarium oxysporum*	[90]

**Table 2:** Common fusion tags for recombinant protein purification.

Protein	Tag Name	Size (kDa)	Length (aa)	Matrix	References
Hexahistidine	His-tag (6x)	1	6–10	Immobilized metal ions (Ni^2+^, Co^2+^, Cu^2+^, Zn^2+^, Fe^3+^)	[39,114]
His-patch thio-fusion	HP- thioredoxin	11.7	100	Metal chelating agents	[115]
Glutathione S-transferase	GST	26	211	Glutathione	[116]
Maltose-binding protein	MBP	42	396	Amylose	[117]
Calmodulin-binding peptide	CBP	2	26	Ca^2+^ chelating agents	[118,119]
Intein-Chitin binding domain	CBD	5.6	51	Chitin	[120]
FLAg tag peptide	FLAG	1.01	8	Anti-FLAG mAb	[121]
Streptavidin/Biotin-binding peptide	SBP	4.3	38	Streptavidin	[119]
Strep-tag peptide	Strep-II	1.06	8	Strep-Tactin	[119]
Halo protein tag	Halo	33	297	Halo-link resin	[122]
Fasciola hepatica8-Da antigen	Fh8	8	69	EDTA	[123]
Myc protein	c-Myc	1.2	11	Anti-Myc epitope mAb	[124]
S protein	S	1.75	15	S-protein RNase A	[125]

**Table 3:** Common proteases for tag removal.

Protease	Source	Cleavage Sites	Molecular Weight (kDa)	References
TEV	Tobacco Etch Virus	ENLYFQ-	27	[39,70]
Enterokinase	*E. coli*	DDDDK-	31	[128]
Factor Xa	Bovine plasma	IDGR-	42 + 17	[129]
Genenase	*Bacillus amyloliquefaciens*	PGAAHY-	28	[130,131]
Thrombin	Bovine plasma	LVPR-GS	6	[132]
PreScission	Human rhinovirus (HRV) 3C	LEVLFQ-GP	46	[133]
Furin	*Spodoptera frugiperda* (Sf9) cells	RXK/RR-	52.7	[134]
Sortase A	*Staphylococcus aureus*	LPET-G	12	[135]
Intein	*S. cerevisiae* (vma gene)	Self-cleavable	51	[136,137]
SUMO	*E. coli*	Conformation (Requires His-tag)	12	[138]
TVMV	Tobacco vein mottling virus	ETVRFQ-S	77.9	[139]
TAGZyme system	*S. frugiperda* (baculovirus)	Exoproteolysis	23 + 16 + 6	[140]
Exoproteases Carboxypeptidase A	Pancreas *E. coli* *S. cerevisiae*	C-terminal amino acids except Pro, Lys, and Arg	33	[140]
Carboxypeptidase B	Pancreas *E. coli* *P. pastoris*	C-terminal Lys and Arg	35	[140,141]
CasP6	*E. coli*	VEID-	26	[142]

(-) The hyphen is used to denote the endo-protease cleavage site.

## Data Availability

Not applicable.

## Supplementary Materials

The following are available online at https://www.mdpi.com/article/10.3390/ijms22168712/s1.
