# Supplementary material for: Antimicrobial Peptides from Plants: A cDNA-Library Based Isolation, Purification, Characterization Approach and Elucidating Their Modes of Action
Source: Int J Mol Sci. 2021 Aug 13;22(16):8712. doi: 10.3390/ijms22168712 (PMC8395713; doi:10.3390/ijms22168712)
Supplement: Supplementary file 1 [file ijms-22-08712-s001.zip › ijms-1280177-supplementary.pdf]

Antimicrobial peptides from plants: a cDNA-library based isolation, purification, characterization approach and elucidating their mode of action

Md. Samiul Islam, Gamarelanbia Mohamed, Shakil Ahmed Polash, Md. Amit Hasan, Razia Sultana, Noshin Saiara, and Wubei Dong

Table S1 Primers and sequences for building the cDNA library

Primer	Sequence
Oligo dT (contains *Xba* I enzyme site)	ACAGGCTCTAGAGCTTTTTTTTTTTTTTT TTTTTTTT
Adapter 1 (contains *Nde* I cleavage site)	CTCGAGAGGAATTCCATATGC
Adapter 2 (contains *Nde* I cleavage site)	GCATATGGAATTCCTCTCGAGTACG
Adapter 3 (contains *Nde* I cleavage site)	CTCGAGAGGAATTCCATATGCT
Adapter 4 (contains *Nde* I cleavage site)	AGCATATGGAATTCCTCTCGAGTACG
Adapter 5 (contains *Nde* I cleavage site)	CTCGAGAGGAATTCCATATGCTA
Adapter 6 (contains *Nde* I cleavage site)	TAGCATATGGAATTCCTCTCGAGTACG
